# Interest in Treatment with GLP-1 Receptor Agonists for the Management of Insufficient Weight Loss or Weight Regain After Bariatric Surgery

**DOI:** 10.1007/s11695-025-08210-y

**Published:** 2025-09-06

**Authors:** Hussein Abdallah, Wissam Hadi Klink, Joseph Derienne, Cosmin Voican, Gabriel Perlemuter, Rodi Courie, Ibrahim Dagher, Hadrien Tranchart

**Affiliations:** 1 Department of Minimally Invasive Digestive Surgery, Antoine Béclère Hospital, AP-HP, 157 Rue de La Porte de Trivaux, 92141 Clamart, Clamart, France; 2 Department of Hepato-Gastroenterology and Nutrition, Antoine Béclère Hospital, AP-HP, 92140 Clamart, Clamart, France

## Abstract

**Background:**

Bariatric surgery (BS) is the most effective treatment for severe obesity, but a significant proportion of patients experience insufficient weight loss (IWL) or weight regain. Glucagon-like peptide-1 receptor agonists (arGLP-1) have emerged as a promising adjunctive therapy for managing these suboptimal outcomes. This study evaluates the efficacy and safety of arGLP-1 in patients with IWL or WR after BS.

**Methods:**

A retrospective analysis was conducted on 100 patients who underwent BS (96 sleeve gastrectomy, 4 gastric bypass) and received arGLP-1 therapy (semaglutide or dulaglutide) for IWL (defined as < 50% excess weight loss (EWL) from baseline), and WR (a ≥ 10 kg increase from the nadir weight post-surgery). Data on weight loss, comorbidities, and adverse events were collected over a median follow-up of 1 year. Statistical analyses included paired *t*-tests, Wilcoxon signed-rank tests, and chi-squared tests.

**Results:**

At 1 year, patients achieved significant weight loss with a median total weight loss (%TWL) of 25.5% and a median excess weight loss (%EWL) of 66.3% compared to 16.6% and 40.8%, respectively, at treatment initiation with BMI reduction of 3.7 kg/m^2^. Significant improvements were observed in comorbidities, including reductions in obstructive sleep apnea (− 30%), hypertension (− 40%), and arthralgia (− 56.5%). Glycated hemoglobin levels decreased by 0.8 points. Treatment was well-tolerated, with nausea being the most common side effect (5% discontinuation rate).

**Conclusion:**

arGLP-1 are effective and safe for managing IWL or WR after BS, leading to significant weight loss, comorbidity improvement, and sustained %TWL. These findings support their use as a valuable adjunctive obesity management medication (OMMs) in post-bariatric care, though long-term adherence and cost-effectiveness require further investigation.

**Graphical Abstract:**

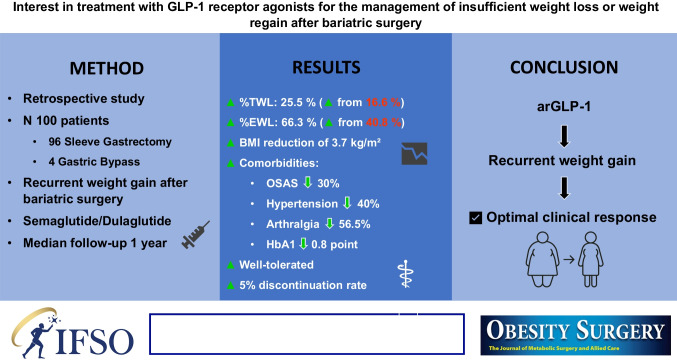

## Introduction

Obesity is an escalating global health issue. Despite extensive preventive efforts, over one billion people worldwide were affected by obesity in 2022 [1]. At present, bariatric surgery (BS) is considered the benchmark treatment of morbid obesity, with well-established indications providing effective long-term outcomes [2, 3] that lead to sustained weight loss [4] and improvement in most weight-related comorbidities [5–7], with relatively low morbidity and mortality [8–10].

Although bariatric surgery is the most effective treatment, a significant proportion of patients experience suboptimal outcomes due to insufficient weight loss (IWL) or weight regain (WR) after surgery. Although multiple definitions of IWL and WR exist in the literature [11, 12], a universally accepted standard is still debated. For the purposes of this study, we adopted the following operational criteria: IWL was defined as < 50% excess weight loss (EWL) from baseline, and WR as a ≥ 10 kg increase from the nadir weight after bariatric surgery. These thresholds were chosen to ensure consistency in patient selection and comparability with previous reports.

IWL and WR are two entities that represent a substantial challenge for bariatric surgeons. Within 5 years post-surgery, WR rates can range from 37% to 76% among patients undergoing gastric bypass [13] and sleeve gastrectomy [14, 15], respectively. Furthermore, a national cohort study found that a notable percentage of patients (up to 10%) required revisional surgery within 5 years of follow-up [16]. Alarmingly, some individuals undergo multiple additional bariatric procedures, including conversional or revisional surgeries [17], to address these outcomes. Recognizing the need for effective strategies to mitigate these challenges in the postoperative period, anti-obesity pharmacotherapy has emerged as a promising adjunctive approach.

Incretin analogs, a class of neurohormones that act in a multimodal way, represent some of the most advanced options in anti-obesity pharmacotherapy. Specifically, glucagon-like peptide 1 receptor agonists (arGLP-1), such as semaglutide, have demonstrated a mean weight loss approaching the 15% threshold [18–20]. More recently, innovative molecules like the dual glucose-dependent insulinotropic polypeptide and the glucagon-like peptide 1 (GLP1R-GIPR) receptors agonists, such as tirzepatide, have shown even greater efficacy, with weight loss up to 18% [21]. Retatrutide, a triple agonist targeting gastric inhibitory polypeptide (GIP), GLP-1, and glucagon receptors, has achieved highly promising outcomes in its phase 2 trial [22], with weight loss up to 24%. This exceptional result highlights its potential as one of the most effective anti-obesity therapies in development, underscoring the therapeutic promise of multi-receptor agonists in weight management. Emerging advancements in anti-obesity treatments include the development of oral formulations [23] and once-monthly [24] dosing regimens, which aim to improve patient adherence and convenience, simplifying the integration of these therapies into daily life.

The range of indications for these drugs is broad and often debated. Their use can be proposed as alternatives for patients who may not undergo surgery, as a bridge to optimize outcomes before or in association with surgery. Nevertheless, it was demonstrated that inadequate weight loss after BS is often associated with increased appetite and an unfavorable postoperative gut hormone profile, including reduced circulating GLP-1 levels [25, 26]. This suggests that treatment with arGLP-1 could be particularly beneficial for individuals struggling with suboptimal weight loss outcomes after surgery. Thus, our department has been a pioneer in utilizing these drugs since the early evidence demonstrated their efficacy. The aim of this retrospective study is to report and analyze the results of the use of arGLP-1 in patients experiencing insufficient weight loss or weight regain after bariatric surgery.

## Materials and Methods

### Study Design and Setting

This retrospective study utilized prospectively collected data from patients treated at our bariatric surgery center between January 2019 and October 2023. The study was conducted in accordance with the ethical standards outlined in the Declaration of Helsinki and was approved by the institutional review board.

### Patient Selection

Patients included in the study had previously undergone either sleeve gastrectomy or gastric bypass and met the following criteria: insufficient weight loss (defined as < 50% excess weight loss from baseline) or weight regain (defined as ≥ 10 kg increase from the nadir weight post-surgery) were included at least 2 years after their initial bariatric surgery.

Patients were initially eligible for surgery if they had a BMI of 40 kg/m^2^ or higher, or a BMI between 35 and 40 kg/m^2^ with significant comorbidities according to French guidelines [27].

Patients received arGLP-1 based on availability and clinical appropriateness: semaglutide was administered via weekly subcutaneous injections, starting at 0.25 mg/week and titrated to a maximum dose of 1 mg/week, while dulaglutide was administered at a fixed dose of 1.5 mg/week.

Demographic, clinical, and surgical data, including age, gender, pre-surgical BMI, type of surgery performed, baseline and nadir weight, comorbidities at baseline and during follow-up, as well as details of arGLP-1 (duration and dosing), were obtained.

Clinical assessments were conducted every 3 months to evaluate efficacy, measured as percentage of total weight loss (%TWL), excess weight loss (%EWL), changes in BMI, and improvement in comorbidities, as well as tolerance, monitored for adverse effects including gastrointestinal symptoms (e.g., nausea, vomiting, diarrhea) and discontinuation rates.

### Statistical Analysis

Descriptive statistics were used to summarize baseline characteristics and outcomes. Continuous variables were reported as means ± standard deviation or medians with interquartile range (IQR), as appropriate. Categorical variables were expressed as frequencies and percentages. Differences between baseline and follow-up measures were analyzed using paired *t*-tests or Wilcoxon signed-rank tests for continuous variables and chi-squared or Fisher’s exact tests for categorical data. Statistical significance was set at *p* < 0.05.

## Results

### Study Population

The study included 100 patients treated with arGLP-1 between January 2019 and October 2023. Of these, 84% were women, with a median BMI of 42 kg/m^2^ at the time of bariatric surgery. Regarding type of surgery, 96% (*n* = 96) had undergone sleeve gastrectomy and 4% (*n* = 4) distal Roux-en-Y gastric bypass. Additionally, 29% had diabetes requiring treatment before surgery. Patient characteristics at baseline, treatment initiation, and after 1 year of follow-up are presented in Table 1.

**Table 1:** Patients’ characteristics at baseline, treatment initiation, and after 1 year of follow-up

**Characteristics**	**Pre-surgery**	**At treatment initiation (*N* = 100)**	**1-year follow-up (*N* = 56)**	***p*-value**
Gender (female/male)	84/16			
Age, y, median (IQR)	48 (40–53)	52 (44–57)	53 (45–58)	
Weight, kg, median (IQR)	114 (102–129)	98.3 (86–110)	86.6 (75–97)	< 0.001
BMI, kg/m^2^, median (IQR)	42 (38.2–45.8)	34.9 (32.0–38.6)	31.2 (28.3–34.4)	< 0.001
%TWL, median (IQR)		16.6 (10.5–24.5)	25.5 (20.4–31.6)	< 0.001
%EWL, median (IQR)		40.8 (27.9–52.7)	66.3 (44.6–116.3)	< 0.001
Co-morbidities				
Type 2 diabetes mellitus, *n* (%)	29/100 (29%)	11/100 (11%)	9/56 (Δ–18.2%)	0.002
Hypertension, *n* (%)	37/100 (37%)	30/100 (30%)	18/56 (Δ–40%)	< 0.001
OSAS, *n* (%)	58/100 (58%)	20/100 (20%)	14/56 (Δ–30%)	0.001
Dyslipidemia, *n* (%)	21/100 (21%)	11/100 (11%)	7/56 (Δ–36.4%)	0.003
Arthralgia, *n* (%)	33/100 (33%)	23/100 (23%)	10/56 (Δ–56.5%)	< 0.001
Fatty liver disease, *n* (%)	29/100 (29%)	22/100 (22%)	14/56 (Δ–36.4%)	0.002
Glycated hemoglobin A1c		6.9 (6.2–7.5)	6.1 (5.7–6.7)	< 0.001

*y* year, *TWL* total weight loss, *EWL* excess weight loss, *OSAS* obstructive sleep apnea syndrome, *IQR* interquartile range, *Δ* delta *(*indicates relative percentage change from treatment initiation to 1-year follow-up)

At treatment initiation, the median BMI was 34.9 kg/m^2^ (IQR: 32.0–38.6) and the median %EWL was 40.6% (IQR: 27.9–52.7). Eleven percent had treated diabetes, 20% were on therapy for obstructive sleep apnea (OSAS), 30% were treated for hypertension, 11% had managed dyslipidemia, 23% reported arthralgia, and 22% had hepatopathy. The median interval between the bariatric procedure and the initiation of pharmacological treatment was 5 years (IQR: 2–7 years).

Of the 100 patients initially enrolled in the study, 11 were classified as dropouts due to being lost to follow-up, while 4 patients discontinued treatment—one due to hypoglycemia, one due to lack of efficacy, and two due to challenges in accessing the medication—resulting in 85 patients completing therapy until the study’s conclusion.

Overall tolerability of the treatment was good, with the most common side effect being nausea, particularly during treatment initiation or dose adjustments, while diarrhea was reported by 5% of patients, which was dose-dependent and easily managed.

The median follow-up duration was 1 year (IQR: 0.5–2 years), with key findings including a median weight loss of 11.7 kg and a median BMI at the most recent follow-up of 31.2 kg/m^2^, reflecting a median reduction of 3.7 kg/m^2^.

Among the 56 patients with complete follow-up data at 1 year, there was a significant improvement in weight-related comorbidities, including a reduction in obstructive sleep apnea syndrome (− 30%), hypertension (− 40%), arthralgia (− 56.5%), and fatty liver disease (− 36.4%). Additionally, there was an average improvement in glycated hemoglobin levels of 0.8 point (± 0.6), highlighting the positive impact of the therapy.

**Fig. 1:**
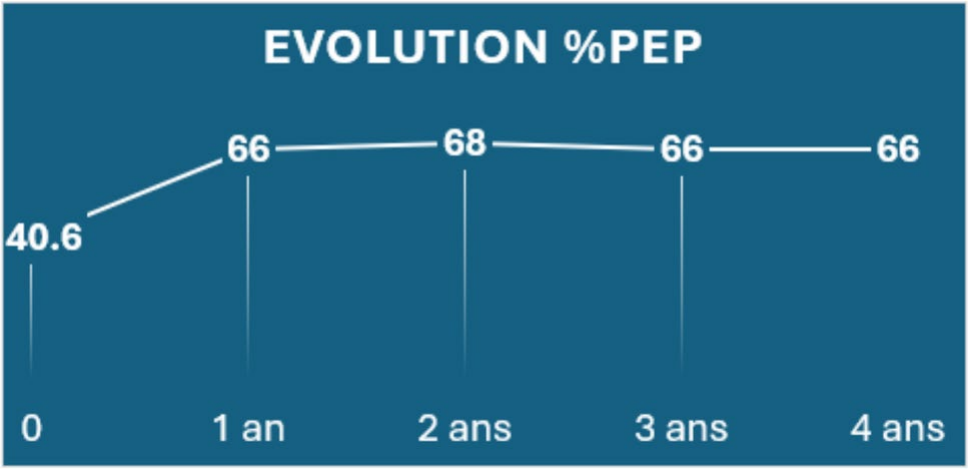
Evolution of %PEP (percentage of excess weight loss) during follow-up. Data points show progressive improvement from pre-treatment baseline (40.6%) to 66–68% with plateau effect maintained through 4 years post-treatment. Data beyond 1 year represent only the subset of patients who maintained therapy and follow-up to that time point

The data show that patients achieved a median %TWL of 25.5% (IQR 20.4–31.6) and %EWL of 66.3% (IQR 44.6–116.3) after 1 year of arGLP-1 treatment, compared to 16.6% (IQR 10.5–24.5) and 40.8% (IQR 27.9–52.7) at the initiation of therapy. Patients who continued treatment with arGLP-1 sustained their excess weight loss over time, with positive outcomes observed even up to 3–4 years of continuous therapy. Data beyond 1 year represent only the subset of patients who maintained therapy and follow-up to that time point (Fig. 1.)

## Discussion

Our study demonstrates that arGLP-1 are a highly effective and well-tolerated option for managing IWL, WR, and improvement of comorbidities following bariatric surgery. Patients treated with arGLP-1 achieved a median weight loss of 11.7 kg, an increase in %TWL of 8.9% and 25.5% in %EWL, respectively, after 1 year of therapy. These results align with prior studies, such as the BARI-OPTIMISE trial [28], which reported a 9.4 kg weight loss with liraglutide in a similar population. Additionally, a systematic review by Dréant et al. [29] highlighted weight loss ranging from 5 to 17% with arGLP-1 in similar contexts, emphasizing the efficacy of this class of drugs in mitigating insufficient surgical outcomes.

Our cohort demonstrated marked improvement in weight-related comorbidities, including reductions in obstructive sleep apnea syndrome (− 30%), hypertension (40%), arthralgia (− 56.5%), and fatty liver disease (− 36.4%). These findings are consistent with prior studies that have documented the metabolic and cardiovascular benefits of arGLP-1, including reductions in glycated hemoglobin levels and blood pressure. The ability of these medications to modulate appetite and energy expenditure—partly through delayed gastric emptying [25] and enhanced central satiety signaling [26]—may account for these favorable outcomes.

The tolerability profile of arGLP-1 in our study was favorable, with transient nausea being the most common side effect, primarily occurring during treatment initiation and dose titration. Consistent with findings from the OASIS 1 trial [23], only 5% of patients discontinued treatment due to gastrointestinal intolerance. While nausea typically resolved over time, diarrhea (observed in 5% of our cohort) tended to persist when present, though it was manageable in most cases with antidiarrheal agents. These findings align with larger clinical trials. Wilding et al. [30] (STEP trials) reported a 44–48% incidence of nausea with semaglutide, but discontinuation rates remained low (< 5%) due to the transient and mild nature of symptoms. The SUSTAIN-4 trial [31] noted persistent diarrhea in 11–13% of semaglutide users, though severe cases were rare (< 2%). Pratley et al. [32] (PIONEER trials) further highlighted that diarrhea was dose-dependent and often controllable with loperamide. Collectively, our results and existing literature support the long-term feasibility of arGLP-1 therapy when side effects are proactively managed.

Despite these promising results, several challenges remain. Long-term adherence and cost-effectiveness are critical issues that may limit the widespread adoption of arGLP-1. The high monthly cost of these medications poses a significant barrier, particularly in resource-limited settings. Furthermore, these treatments are not yet reimbursed by social security health system in France. While our study demonstrated sustained weight loss over a median follow-up of 1 year, longer-term data are needed to assess the durability of these outcomes. Future studies should also explore the potential of emerging therapies, such as dual and triple agonists (e.g., tirzepatide and retatrutide), which have shown even greater efficacy in weight loss [20, 21].

Our cohort represents a specific subgroup of post-bariatric patients with IWL or WR who required pharmacological intervention. These patients may differ in baseline characteristics, behavioral factors, or comorbidity burden compared with those achieving optimal surgical outcomes, introducing potential selection bias inherent to retrospective designs. The absence of a control group precludes definitive conclusions on causality between GLP-1 receptor agonist therapy and observed outcomes, and residual confounding cannot be excluded

In conclusion, arGLP-1 represent a valuable addition to the therapeutic arsenal for managing IWL or WR after BS. They offer significant weight loss, comorbidity improvement, and a manageable safety profile. However, these results should be interpreted considering the study’s limitations, including its retrospective design, the absence of a control group, and the potential for selection bias. Addressing barriers such as cost, long-term adherence, and patient selection criteria will be essential to optimize their integration into clinical practice. Larger, randomized controlled trials with extended follow-up periods are needed to establish definitive guidelines for the use of arGLP-1 in post-bariatric care.

## Data Availability

No datasets were generated or analyzed during the current study.
